# Supplementary material for: Detecting ancient positive selection in humans using extended lineage sorting
Source: Genome Res. 2017 Sep;27(9):1563–72. doi: 10.1101/gr.219493.116 (PMC5580715; doi:10.1101/gr.219493.116)
Supplement: Supplemental Material [file supp_27_9_1563__index.html]

Detecting ancient positive selection in humans using extended lineage sorting — Supplemental Material 

# Detecting ancient positive selection in humans using extended lineage sorting

## Supplemental Material

- Supplemental\_Material.docx
- Supplemental\_Files\_1-3.zip
- Supplemental\_Code.zip
