# Supplementary material for: Detecting ancient positive selection in humans using extended lineage sorting
Source: Genome Res. 2017 Sep;27(9):1563–72. doi: 10.1101/gr.219493.116 (PMC5580715; doi:10.1101/gr.219493.116)
Supplement: Supplemental Material [file supp_gr.219493.116_Supplemental_Material.docx]

**Supplemental Material:**

**Detecting ancient positive selection in humans using extended lineage sorting**

Stéphane Peyrégne^*^, Michael James Boyle, Michael Dannemann, Kay Prüfer^*^

Department of Evolutionary Genetics, Max Planck Institute for Evolutionary Anthropology, 04103 Leipzig, Germany.

*Corresponding authors: stephanepeyregne@gmail.com; pruefer@eva.mpg.de

# TABLE OF CONTENTS:

**Supplemental Methods** (p3-5)

**Supplemental Information 1:** Demographic parameters used in coalescent simulations. (p6)

**Supplemental Information 2:** Examples of command lines for some simulations. (p6-7)

**Supplemental Figure S1:** Estimation accuracy of emission probabilities for the internal state. (p8)

**Supplemental Figure S2**: Estimation accuracy of one error parameter. (p9)

**Supplemental Figure S3**: Estimation accuracy of the average length of internal and external regions using a 2-state hidden Markov model. (p10)

**Supplemental Figure S4**: Receiver Operating Characteristic curves for labelling sites with the correct states. (p11)

**Supplemental Table S1**: Performance of the Hidden Markov Model to detect selection in humans. (p12)

**Supplemental Table S2:** Support for the 3-state model with ELS regions compared to a 2-state model without those regions. (p13)

**Supplement Table S3:** Comparison of posterior probabilities for the ELS state in low-ILS regions in apes and the remaining regions on the X Chromosome. (p13)

**Supplement Table S4**: Age distribution comparison between selective events detected by the ELS HMM and different methods. (p13)

**Supplemental Table S5**: Overlap between candidates from ELS signals and previous scans for ancient selection. (p13)

**Supplemental Table S6**: Expression enrichment analysis for genes in the extended set of candidate regions. (p14)

**Supplemental Table S7**: Expression enrichment analysis for genes in the vicinity of the extended set of candidate regions. (p14)

**Supplemental Figure S5:** Average Neanderthal ancestry around candidates for selection (based on the African-American map). (p15)

**Supplemental Figure S6:** Average Neanderthal ancestry around candidates for selection (based on the deCODE map). (p16)

**Supplemental Table S8:** Genes from the core set of candidate regions overlapping with long deserts of Neandertal and Denisovan ancestry. (p17)

**Supplemental Figure S7**: Overlap with candidate regions from previous ancient sweep screens. (p17)

**Supplemental Table S9:** Comparison of past and current recombination rates (RR) for each candidate regions in the core set. (p18-19)

**Supplemental Figure S8**: Comparison of the allele sharing between Neandertal and Modern humans in simulations and real data. (p20)

**Supplemental Table S10**: Performance of the Hidden Markov Model for different split times (conservative cutoffs). (p21)

**Supplemental Table S11**: Performance of the Hidden Markov Model for different split times (permissive cutoffs). (p21)

**References** (p22)

**Supplemental Methods**

## Basic processing

We used single nucleotide polymorphisms (SNPs) from 185 unrelated Luhya and Yoruba individuals from the 1000 Genomes Project phase I (The 1000 Genomes Project Consortium 2012), corresponding to 370 sets of autosomes and 279 X Chromosomes. In order to add sites where all Africans differ from the common ancestor with chimpanzee, we first compiled a list of all sites where six high-coverage African genomes (Mbuti, San and Yoruban A and B-panel individuals from Prüfer et al. 2014) are identical. A site was regarded fixed different when the whole genome alignments of at least three out of four ape reference genome assemblies (chimpanzee (panTro3) (Mikkelsen et al. 2005), bonobo (panPan1.1) (Prufer et al. 2012), gorilla (gorGor3) (Scally et al. 2012) and orangutan (ponAbe2) (Locke et al. 2011); LASTZ (Harris, 2007) alignments to the human genome GRCh37/hg19 prepared in-house and by the UCSC Genome Browser (Speir et al., 2016)) had coverage and were different from the African allele, and when the site was not marked as polymorphic among the 1000 Genomes Luhya and Yoruba individuals.

Neandertal and Denisova alleles at polymorphic and fixed positions were extracted from published VCFs (Danecek et al., 2011) and positions were further filtered to sites passing the published map35_100 filter for both the Denisova and Neandertal genotypes (Prüfer et al., 2014). Sites where either Neandertal or Denisova carried a third allele were disregarded.

Over all autosomes, 11 million SNPs passed the filters in addition to 6.6 million African fixed variants. For the X Chromosome, pseudoautosomal regions, defined as Chr X: 60,001-2,699,520, Chr X: 154,931,044-155,260,560 in hg19 coordinates (http://www.ncbi.nlm.nih.gov/assembly/2758/), were filtered out and around 315,000 SNPs as well as 248,000 African fixed variants remained for analysis.

## Simulations

We simulated sequences using a model of recent human demography to test the performance of our HMM under different scenarios of neutral evolution, positive selection or background selection. Demographic parameters and examples of command lines are shown in Supplemental Information 1 and 2, below.

We generated a total of 100 loci of 1Mb-long sequences under neutrality to investigate the accuracy of labeling external and internal regions using our HMM (see Supplemental Figure S4). To evaluate the length of external regions expected under neutrality for the Chromosome X, we simulated 100 loci of 1Mb-long sequences under the demographic model shown in Supplemental Information 1 with the exception that all effective population sizes were reduced to 75% of the original value. To evaluate the accuracy of parameter estimation, we additionally simulated splits of two populations (including an out-group individual) with a constant population size and different split times ranging from 400ky to 1My (step-size of 50ky). For each condition, we generated 25 sets of 10 Mb each (see Supplemental Figure S3). In an additional set of 100 loci of 1Mb, we introduced random errors by changing the state of the archaic allele with different rates in order to assess our error estimates (see Supplemental Figure S2).

To assess our power to detect events of positive selection, we explored selection coefficients ranging from 0.0005 to 0.1 and different times for the occurrence of the selected allele (every 100ky from 200kya to 600kya) using the coalescent simulator msms (Ewing & Hermisson, 2010) (see Figures 2 and 3; and Supplemental Table S1). The selected mutation was introduced in the middle of the sequence and we assumed an additive effect of the selected mutation (i.e. the homozygous genotype has twice the advantage stated by the selection coefficient). We performed 2000 simulations of 100kb-long loci for which all demographic parameters match our neutral simulations as described in Supplemental Information 1. We used the –SForceKeep switch to drop the simulation if the selected mutation was lost. As 100kb loci are too short to make reliable parameter inferences, we concatenated our simulated sequences, intermittently combining them with 1Mb-long neutral loci from the previous simulations to limit the extent of the sequence affected by positive selection.

To explore the power over different settings of divergence, we simulated a simple demographic model with constant population size and varying degrees of divergence between two populations (see Supplemental Table S10 and S11 for further details).

We investigated how background selection affects lineage sorting in and around a conserved region by performing forward in time simulations using SLiM (Messer, 2013). The simulated locus of 500kb length contained a conserved region resembling an ‘average’ human gene (see pg. 19 of the documentary accompanying SLiM (Messer 2013)) and covered 100kb (20%) of the simulated locus (see Supplemental Information 2). Mutations in the conserved region were assumed to be neutral (25%) or deleterious (75%), with the selection coefficients of the deleterious mutations drawn from a gamma distribution with mean s = −0.05 and shape parameter α = 0.2. The deleterious mutations were assumed to be partially recessive with dominance coefficient h = 0.1 for a set of 100 simulations. To explore the effect of the strength of selection on the results, we produced 2 other sets of 40 simulations each by varying the mean of the gamma distribution (s = -0.001 and -0.1) (see Figure 4).

## Gene Ontology and gene expression analysis

We defined genes that show tissue-specific expression levels using the Illumina BodyMap 2.0 RNA-seq data (Derrien et al., 2012), which contains expression data from 16 human tissues. We computed differential expression for all genes between a given tissue and all other tissues pooled using the DESeq package (Anders & Huber, 2010) and genes were defined to be expressed in a tissue-specific manner when their expression levels were significantly higher (*P*-value < 0.05) in a given tissue compared to all other tissues. We tested for enrichment of candidate genes in the 16 sets of tissue-specifically expressed genes comparing to genes that were located outside of candidate regions using Fisher’s exact test. We calculated family-wise error rates for each tissue by randomly placing regions of sizes similar to the candidate regions in the genome. We repeated this process 1000 times, performed the same enrichment analysis as described above and counted how often any tissue in the randomized sets yields a smaller or equal *P*-value than the *­P-*value observed in the candidate regions for a given tissue. This strategy corrects for the difference in length of genes expressed in specific tissues. We performed a similar analysis for the Gene Ontology analysis using func and the hypergeometric test (Prüfer et al., 2007), again comparing the genes associated with the candidate regions to a thousand sets of random regions to calculate family-wise error rates.

In an attempt to include potential regulatory changes in these enrichment tests, we repeated by assigning genes to candidate regions when a region fell upstream or downstream of a gene (latest Ensembl gene annotation for hg19, release 82 (Aken et al., 2016)). A regulatory region was defined as at least 5kb upstream and 1kb downstream of each gene. The regulatory region was extended until it reached a size of 1Mb or came within 5kb upstream or 1kb downstream of a neighboring gene.

**Supplemental Information 1:** **Demographic parameters used in our coalescent simulations.** We assumed a mutation rate of 1.45 x 10^-8^ per bp per generation and a recombination rate of 1 cM.Mb^-1^. Effective population sizes are reported in thousands of individuals.


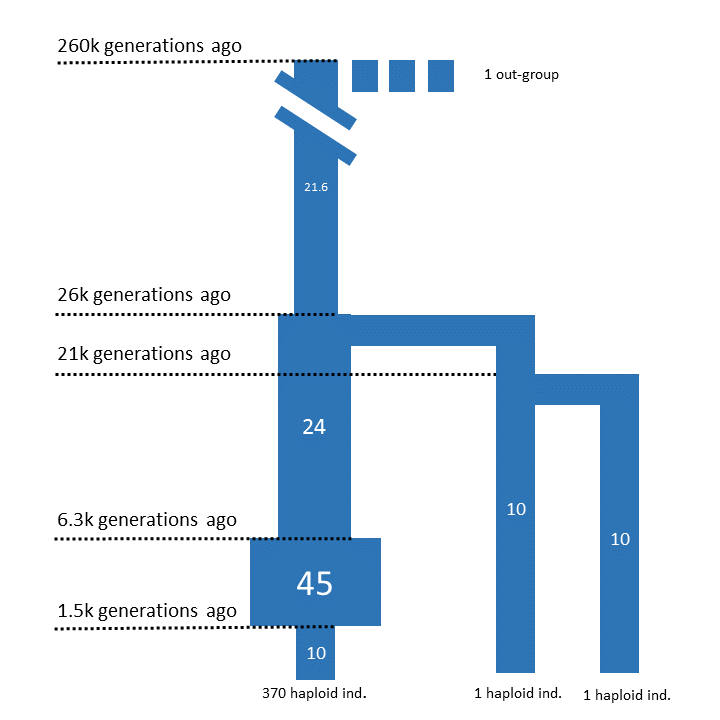


**Supplemental Information 2: Examples of command lines for some simulations.**

**Example of coalescent simulations with scrm** (Staab, Zhu, Metzler, & Lunter, 2014):

scrm 373 100 -T -t 500 -r 400 1000000 -I 4 1 1 1 370 -en 0.0375 4 4.5 -en 0.1575 4 2.4 -ej 0.525 3 2 -ej 0.65 2 4 -en 0.65 4 2.16 -ej 6.5 4 1

**Example of coalescent simulations with positive selection using msms** (Ewing & Hermisson, 2010):

msms 373 2000 -T -N 10000 -t 50 -r 40 100000 -I 4 1 1 1 370 -en 0.0375 4 4.5 -en 0.1575 4 2.4 -ej 0.525 3 2 -ej 0.65 2 4 -en 0.65 4 2.16 -ej 6.5 4 1 -SI 0.6 4 0 0 0 0.00005 -SAA 200 -SAa 100 -Sp 0.5 -Smark -SForceKeep

**Example of parameter file for simulating Background selection with SLiM** (Messer, 2013)**:**

#MUTATION TYPES

m1 0.1 g -0.05 0.2 / deleterious (gamma DFE, h=0.1)

m2 0.5 f 0.0 / neutral

#MUTATION RATE

1.45e-8

#GENOMIC ELEMENT TYPES

g1 m1 0.75 m2 0.25 / exon (75% del, 25% neutral)

g2 m2 1.0 / intron (100% neutral)

#CHROMOSOME ORGANIZATION

g2 1 200000 / 1. neutral region

g1 200001 300000 / 2. conserved region

g2 300001 500000 / 3. neutral region

#RECOMBINATION RATE

500000 1e-8

#GENERATIONS

448275

#DEMOGRAPHY AND STRUCTURE

1 P p1 20000 / single population of 20000 individuals

425517 P p3 20000 p1 / Split Archaic - Modern

433448 P p2 20000 p1 / Split Neanderthal - Denisova

#OUTPUT

448275 R p1 1 MS

448275 R p2 1 MS

448275 R p3 370 MS

448275 F


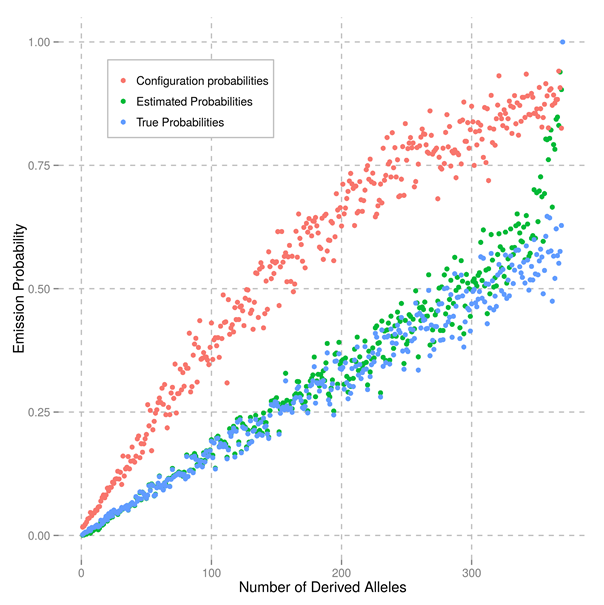


**Supplemental Figure S1:** Estimation of emission probabilities for the internal state. In this state, for each derived allele frequency (here reported as the number of derived alleles in the population), there is a different probability for the archaic lineage carrying also the derived allele. We start the hidden Markov model with arbitrary probabilities (pink dots) and estimate them from the data with the Baum-Welch algorithm. After a few iterations of the algorithm (convergence criteria: 40 iterations maximum or a difference of log-likelhood maxima difference of less than 10^-4^), the estimated probabilities (green dots) converge to the known true probabilities (blue dots). The true probabilities were calculated by counting the fraction of segregating sites in the population (with a given allele frequency) for which the archaic is also derived and look only at those sites in regions where the archaic lineage falls internal to the population variation, according to the coalescent trees provided by the simulator.


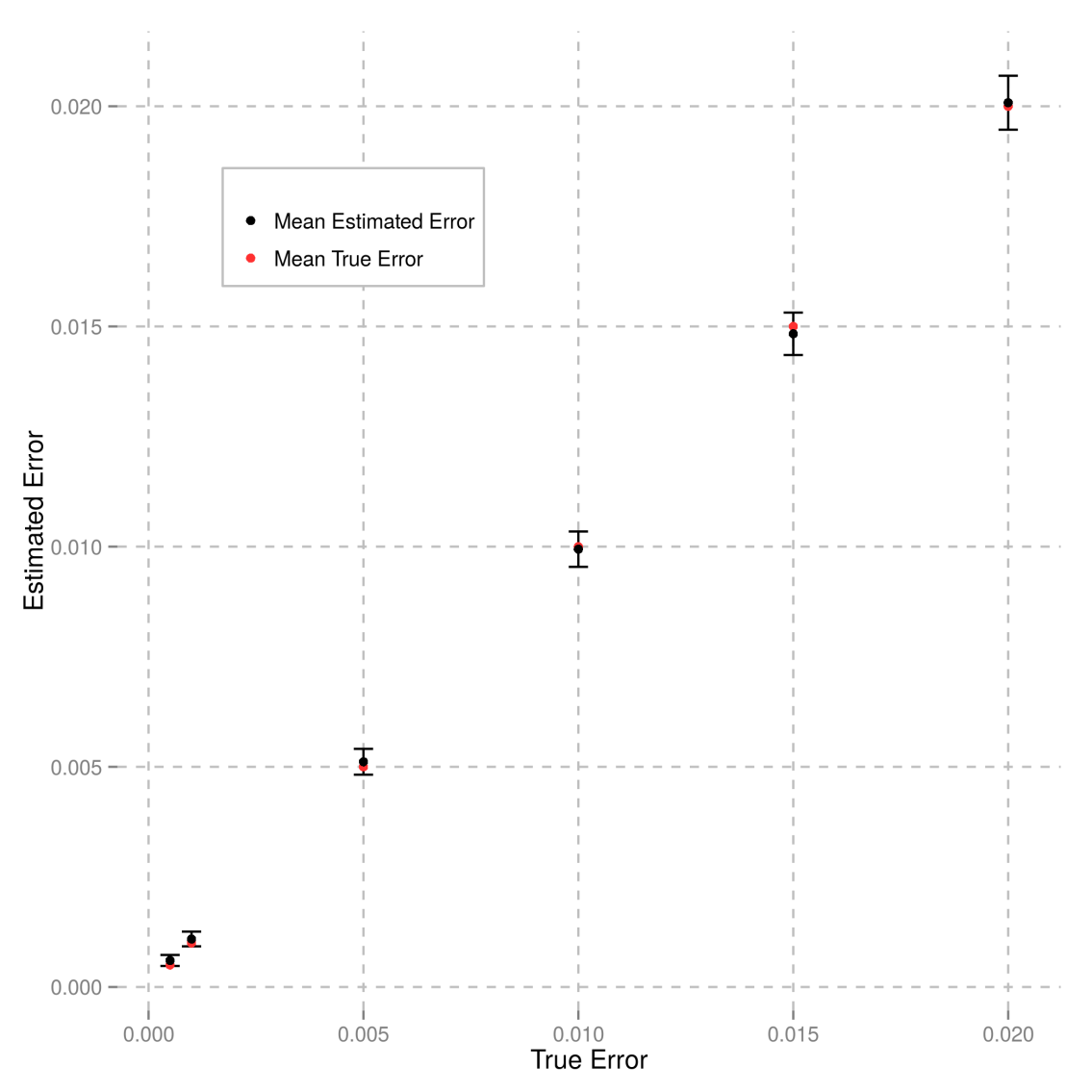


**Supplemental Figure S2**: Estimates of one error parameter of the model. For internal regions, the archaic is expected to share the derived allele at all modern human fixed derived sites, unless the site is erroneously read as ancestral. To test the accuracy of estimating this type of error, we randomly introduced a fixed fraction of ancestral alleles in our simulated archaic genome at sites that are fixed derived in the simulated human population (x-scale). The y-scale shows the estimated error rates, and confidence interval for the estimates were calculated from several sets of simulations.


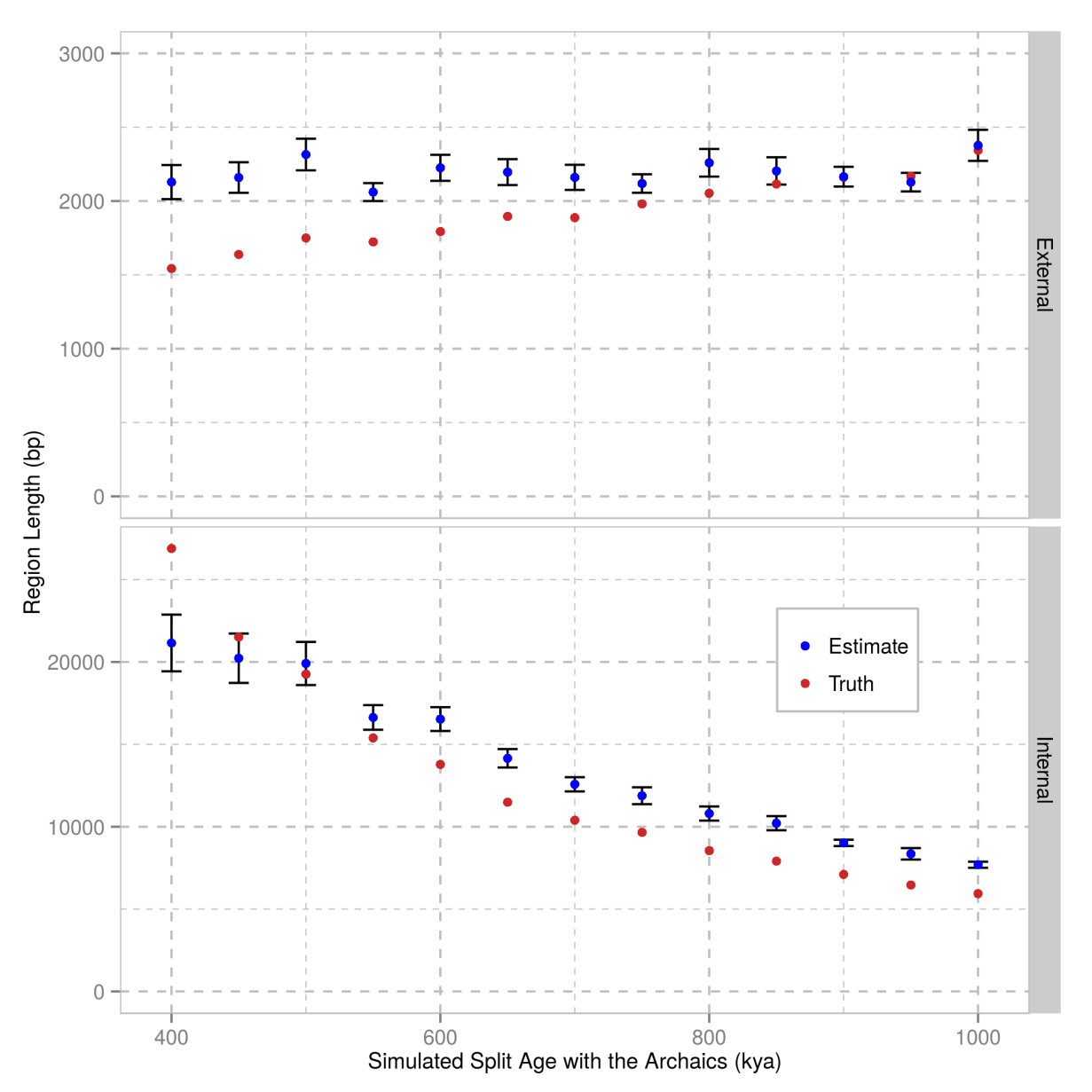


**Supplemental Figure S3**: Estimates of the average length of internal and external regions using our 2-state hidden Markov model which excludes the ELS state. We simulated a split of two populations at varying time points in the past. For this set of simulations, we assumed constant population sizes. Confidence intervals for the estimates were calculated from several sets of simulations.


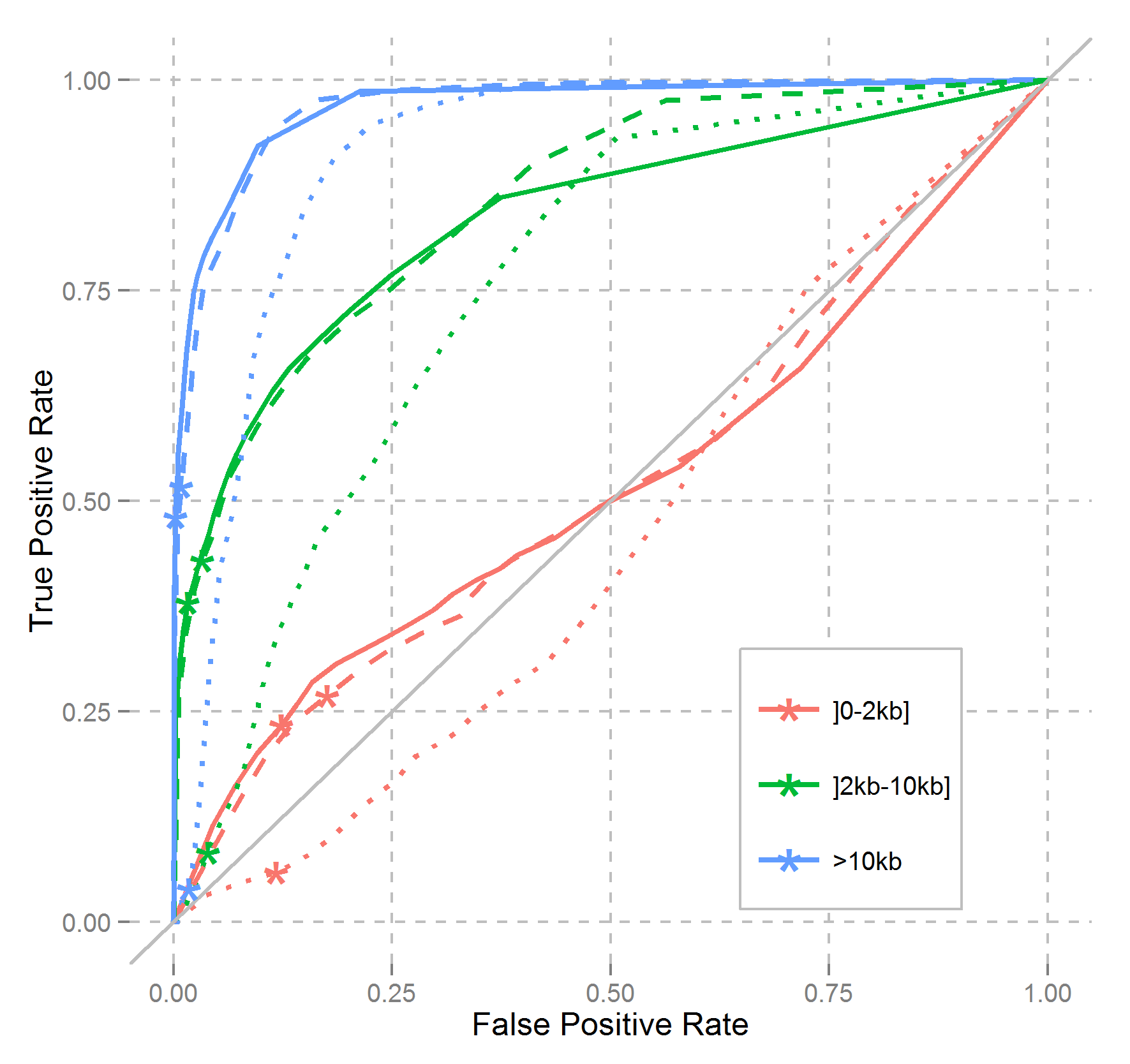


**Supplemental Figure S4**: Receiver Operating Characteristic (ROC) curves for labelling sites in external regions using the estimated parameters (solid lines) or the true parameters used in the simulations (dashed lines). The dotted lines correspond to ROC curves when we remove fixed differences from the analysis. We partitioned the external regions according to their true length (below 2kb, in pink, between 2kb and 10kb, in green, and above 10kb in blue) and plot the corresponding ROC curves. The stars represent the results using a posterior probability cutoff of 0.8 above which sites are labelled external.

**Supplemental Table S1**: Performance of the Hidden Markov Model. We filter the regions according to their length to maximize the specificity for positive selection.

| **Length cutoff** | **Origin of Selection** | **Selection Coefficient** | **True Positive Rate** | **False Positive Rate** |
| --- | --- | --- | --- | --- |
| No cutoff | 300 kya | 0.005 | **0.916** | **0.102** |
| No cutoff | 600 kya | 0.005 | **0.868** | **0.112** |
| >0.025 cM | 300 kya | 0.005 | **0.678** | **0.000** |
| >0.025 cM | 600 kya | 0.005 | **0.651** | **0.002** |

**Supplemental Table S2:** Chi-square p-values from likelihood ratio tests comparing the 3-state model with ELS regions to a 2-state model without those regions. Each test shows a significantly better fit for the 3-state model compared to the 2-state model

| Chromosome | Runs using the deCode map | | Runs using the African-American map | |
| --- | --- | --- | --- | --- |
|  | Altai | Denisova | Altai | Denisova |
| 1  2  3  4  5  6  7  8  9  10  11  12  13  14  15  16  17  18  19  20  21  22 | 9.199e-110  1.393e-144  7.737e-84  1.736e-117  3.673e-122  8.096e-102  1.223e-116  2.826e-68  1.530e-49  4.698e-83  4.866e-70  4.258e-73  1.550e-57  1.467e-49  2.556e-37  1.067e-33  1.470e-36  5.352e-62  6.247e-34  1.509e-48  9.128e-23  1.553e-20 | 3.396e-136  2.013e-145  2.195e-119  5.363e-138  5.227e-97  3.771e-128  4.912e-106  3.104e-89  6.516e-61  3.936e-86  5.542e-85  3.358e-75  1.456e-63  3.559e-86  3.340e-44  1.344e-41  2.543e-33  6.850e-56  2.050e-20  2.209e-36  7.713e-14  2.417e-14 | 1.216e-70  9.906e-109  1.171e-77  5.174e-88  3.527e-70  2.179e-65  1.161e-47  1.060e-55  8.389e-35  2.274e-32  9.311e-53  1.089e-48  9.645e-30  2.850e-25  3.090e-21  1.042e-21  2.547e-37  6.591e-38  1.216e-06  2.238e-23  1.952e-10  1.344e-10 | 3.726e-85  2.934e-84  7.780e-62  4.500e-81  3.150e-65  3.213e-67  2.509e-52  2.113e-46  1.398e-53  2.452e-45  4.727e-58  2.055e-51  7.166e-39  9.768e-46  2.009e-31  8.276e-33  4.687e-45  3.327e-35  4.731e-30  6.320e-23  9.386e-11  3.362e-09 |

**Supplement Table S3:** Comparison of posterior probabilities for the ELS state in low-ILS regions in apes (Dutheil, Munch, Nam, Mailund, & Schierup, 2015; Nam et al., 2015) and the remaining regions on the X Chromosome (*Mann-Whitney U* test, one-sided *P*-values, alternative hypothesis = low-ILS regions exhibit greater posterior probabilities for ELS than in background regions).

|  | **Neanderthal** | **Denisova** |
| --- | --- | --- |
| **African-American map** | < 2.2e-16 | < 2.2e-16 |
| **deCode map** | < 2.2e-16 | < 2.2e-16 |

**Supplement Table S4**: Age distribution comparison between selective events detected by the ELS HMM and different methods. Distributions are significantly different from each other (Kruskal-Wallis rank sum test, *P*-value= 2.11e-09) and the ELS HMM detects significantly older events than the other scans. We report each pair-wise comparison with the ELS results in this table.

| **Selection scan** | **One-sided p-value (Wilcoxon rank sum test)** |
| --- | --- |
| Random sites | < 2.2e-16 |
| Fst | 1.496e-10 |
| iHS | < 2.2e-16 |
| XP-EHH | < 2.2e-16 |
| HKA | 0.002593 |
| CLR | < 2.2e-16 |
| Tajima’s D | < 2.2e-16 |
| Fay and Wu’s H | 0.004071 |
| XP-CLR | < 2.2e-16 |

**Supplemental Table S5**: Overlap between candidates from ELS signals and previous scans for ancient selection (Racimo, 2016; Racimo, Kuhlwilm, & Slatkin, 2014). Focusing on the core set of candidates, a unique region is identified by all methods in the proximity of the ADSL gene. An additional region overlapped STX1A with the extended candidate set.

|  | 3P-CLR: 85 candidates (0.25cM windows) | ABC: 32 candidates |
| --- | --- | --- |
| Core set | 5 (p-value=0.034) | 2 (p-value=0.002) |
| Extended set | 18 (p-value<10^-3^) | 7 (p-value<10^-3^) |

**Supplemental Table S6**: Expression enrichment analysis for genes in the extended set of candidate regions. Odds ratios were obtained from the comparison with the remaining genes in the non-filtered parts of the genome. Family-wise error rates (FWER) were calculated by generating 1000 sets with regions of similar length (randomly placed in the non-filtered parts of the genome for each of those sets) and comparing the Fisher’s test *P*-value of the real set to the lowest *P*-value of each random set.

| Tissue | Odds Ratio | Fisher’s test *P*-value | Empirical *P*-value | FWER |
| --- | --- | --- | --- | --- |
| Adipose | 1.90 | 0.23 | 0.083 | 0.996 |
| Adrenal | 2.50 | 0.13 | 0.078 | 0.984 |
| Blood | 0.71 | 0.87 | 0.594 | 1.00 |
| Brain | 1.60 | 0.015 | 0.648 | 0.721 |
| Breast | 0.00 | 1.00 | 1.00 | 1.00 |
| Colon | 0.00 | 1.00 | 1.00 | 1.00 |
| Heart | 1.80 | 0.099 | 0.28 | 0.958 |
| Kidney | 0.64 | 0.84 | 0.70 | 1.00 |
| Liver | 0.68 | 0.91 | 0.689 | 1.00 |
| Lung | 0.72 | 0.80 | 0.492 | 1.00 |
| Lymph | 0.00 | 1.00 | 1.00 | 1.00 |
| Ovary | 0.00 | 1.00 | 1.00 | 1.00 |
| Prostate | 0.55 | 0.84 | 0.654 | 1.00 |
| Skeletal muscle | 1.10 | 0.46 | 0.379 | 1.00 |
| Testis | 0.45 | 1.00 | 1.00 | 1.00 |
| Thyroid | 1.00 | 0.60 | 0.523 | 1.00 |

**Supplemental Table S7**: Expression enrichment analysis for genes in the vicinity of the extended set of candidate regions. See Material and Methods for how neighboring genes were associated with each region.

| Tissue | Odds Ratio | Fisher’s test *P*-value | Empirical *P*-value | FWER |
| --- | --- | --- | --- | --- |
| Adipose | 1.40 | 0.54 | 0.217 | 1.00 |
| Adrenal | 1.90 | 0.17 | 0.094 | 1.00 |
| Blood | 0.65 | 0.16 | 0.709 | 1.00 |
| Brain | 2.10 | 0.00000085 | 0.335 | 0.336 |
| Breast | 0.78 | 1.00 | 0.643 | 1.00 |
| Colon | 0.00 | 0.035 | 1.00 | 1.00 |
| Heart | 2.10 | 0.01 | 0.15 | 0.977 |
| Kidney | 0.87 | 0.86 | 0.705 | 1.00 |
| Liver | 0.61 | 0.061 | 0.828 | 1.00 |
| Lung | 1.00 | 0.87 | 0.318 | 1.00 |
| Lymph | 0.44 | 0.73 | 0.68 | 1.00 |
| Ovary | 0.00 | 0.18 | 1.00 | 1.00 |
| Prostate | 0.62 | 0.77 | 0.775 | 1.00 |
| Skeletal muscle | 1.20 | 0.31 | 0.114 | 1.00 |
| Testis | 0.68 | 0.072 | 0.915 | 1.00 |
| Thyroid | 0.86 | 1.00 | 0.786 | 1.00 |

**
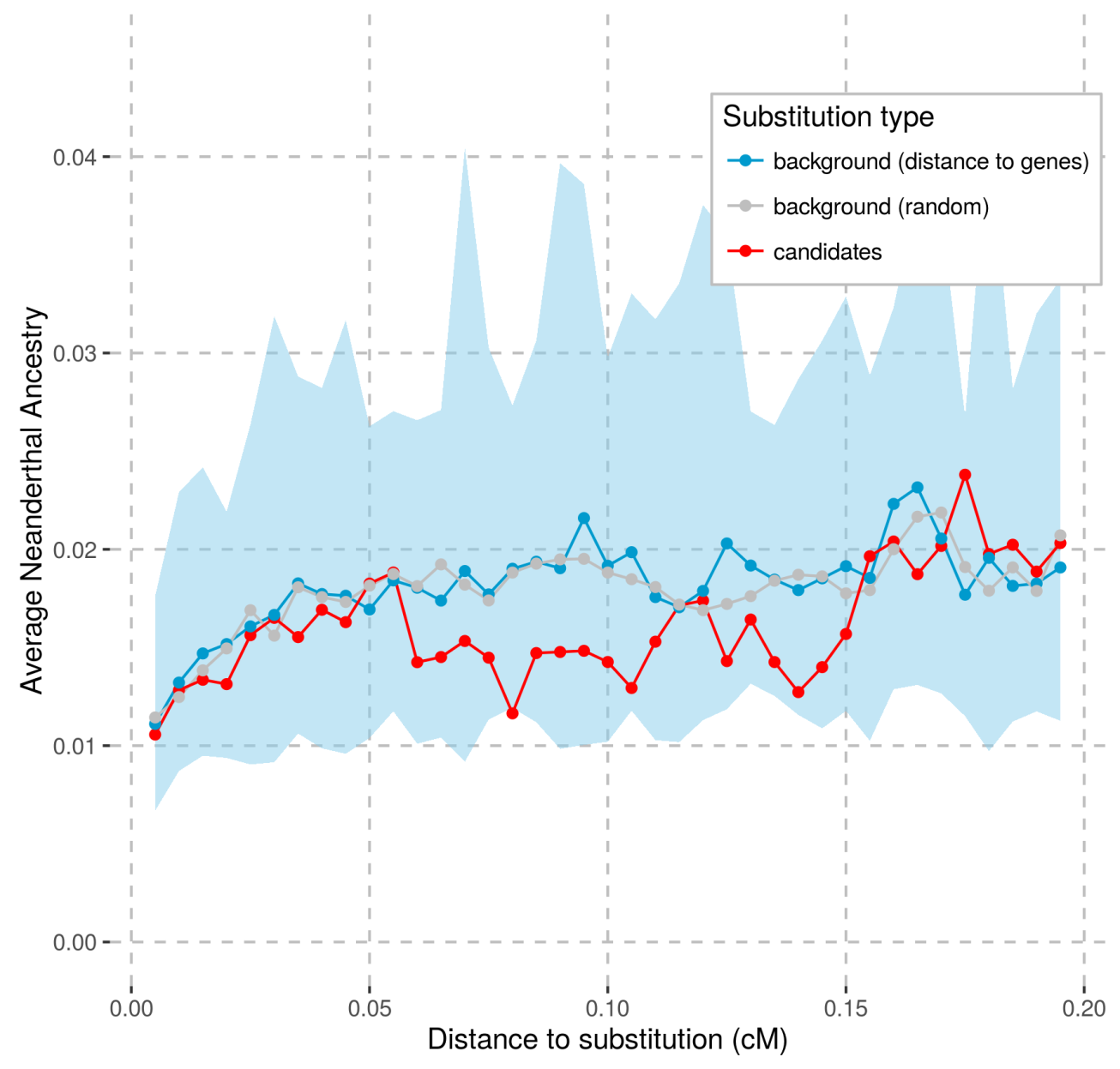
**

**Supplemental Figure S5:** Average Neanderthal ancestry in 0.005 cM sliding windows starting at the fixed differences in the core set of candidate regions and sliding away in both directions. This figure represents results using the African-American recombination map (Hinch et al., 2011). The average Neanderthal ancestry was estimated as the average posterior probability of falling in a Neanderthal haplotype over all segregating sites in the windows using results from (Sankararaman et al., 2014). For each candidate region, only one fixed difference was chosen randomly as the starting point. The blue area corresponds to the 95% confidence interval in the background set of fixed differences chosen randomly but not falling in the candidate regions and matching distances to genes.

**
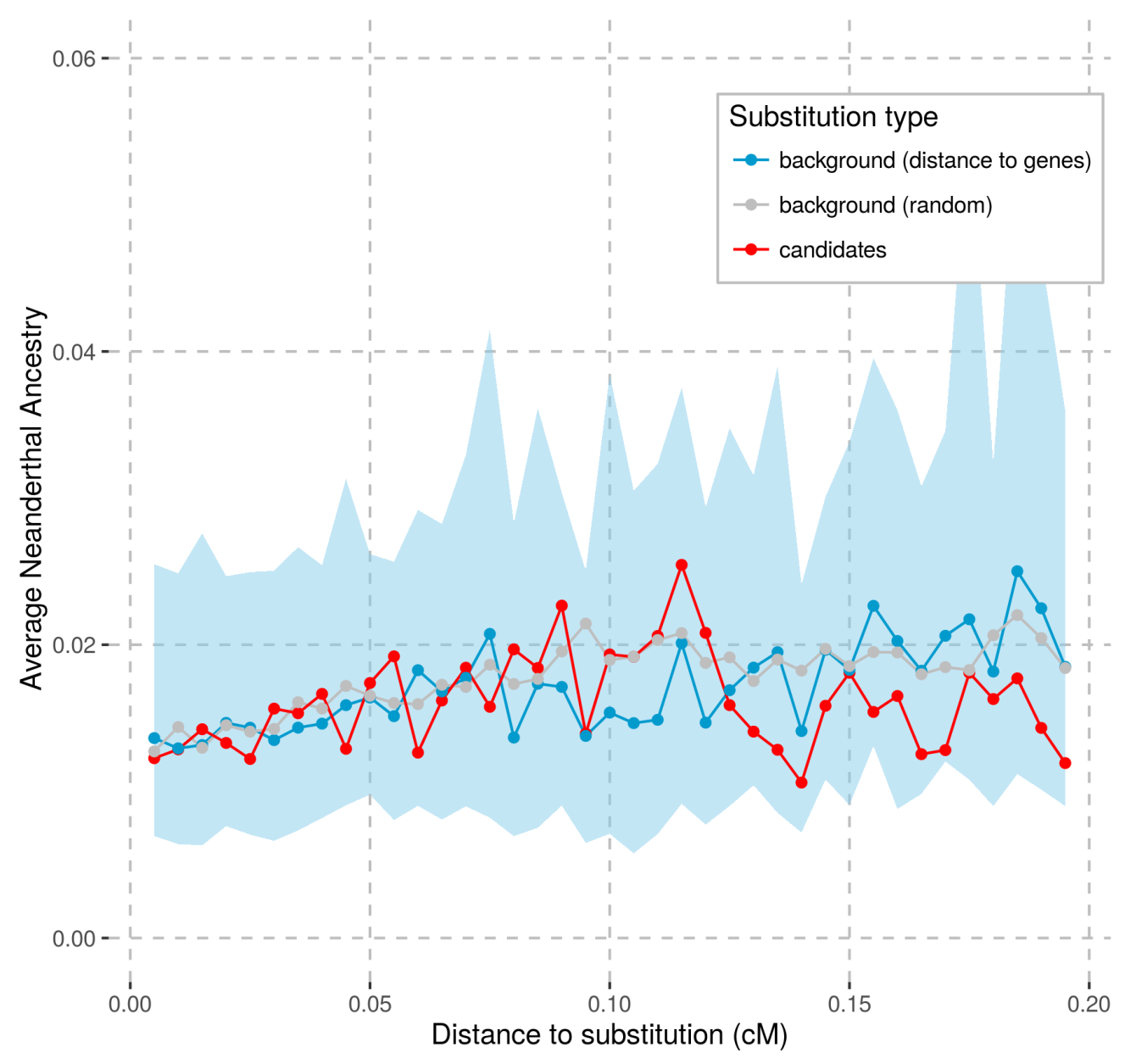
**

**Supplemental Figure S6:** Same analysis as Supplemental Figure S6 using the deCODE recombination map (Kong et al., 2010).

**Supplemental Table S8:** Genes from the core set of candidate regions overlapping with long deserts of Neandertal and Denisovan ancestry.

| Chromosome | Start | End | Overlapping Genes | Overlapping Regulatory Domains |
| --- | --- | --- | --- | --- |
| Chr 1 | 104000000 | 104154236 | *AMY2B, RNPC3* | *COL11A1* |
| Chr 1 | 113429666 | 113560554 | *SLC16A1* | *FAM19A3, LRIG2* |
| Chr 3 | 77027850 | 77033270 | *ROBO2* | *-* |
| Chr 7 | 122320038 | 122379695 | *RNF133, RNF148, CADPS2* | *TAS2R16* |
| Chr 10 | 107809941 | 107866217 | - | SORCS1, SORCS3 |

**
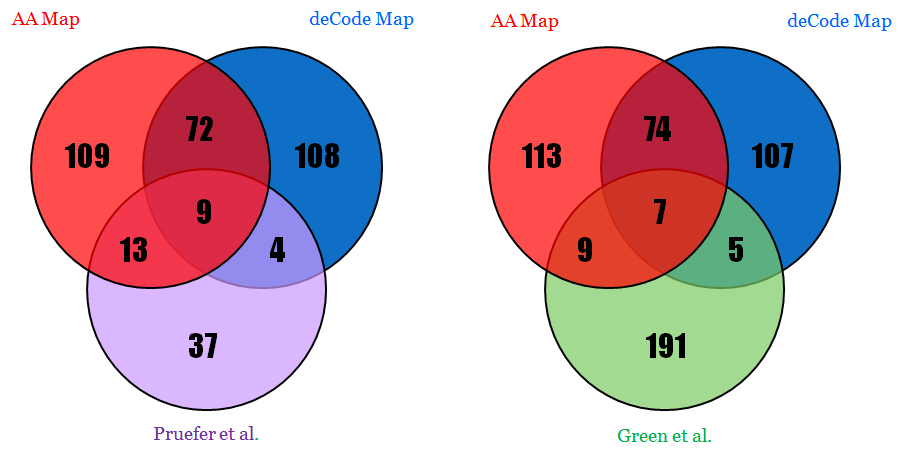
**

**Supplemental Figure S7**: Venn diagram showing the overlap between candidate regions from different sweep screens. Red and blue disks represent the sets of candidates from the scans of this manuscript, using the African-American and deCODE recombination maps respectively. The purple disk corresponds to the 63 top candidate regions reported in a previous implementation of the present hidden markov model (Prüfer et al., 2014).

**Supplemental Table S9:** Comparison of past and current recombination rates (RR) for each candidate regions in the core set. The regions are ranked according to their fold change in recombination rates. Positions are reported in hg19 coordinates and recombination rates in cM/Mb. Past recombination rates were retrieved from (Munch, Mailund, Dutheil, & Schierup, 2014).

| rank | Chromosome | Start | End | Current RR | Past RR | Fold change | Physical length |
| --- | --- | --- | --- | --- | --- | --- | --- |
| 1  2  3  4  5  6  7  8  9  10  11  12  13  14  15  16  17  18  19  20  21  22  23  24  25  26  27  28  29  30  31  32  33  34  35  36  37  38  39  40  41  42  43  44  45  46  47  48  49  50  51  52  53  54  55  56  57  58  59  60  61  62  63  64  65  66  67  68  69  70  71  72  73  74  75  76  77  78  79  80  81 | chr10  chr1  chr13  chr14  chr1  chr17  chr18  chr2  chr2  chr5  chr7  chr8  chr22  chr5  chr7  chr19  chr10  chr16  chr4  chr10  chr1  chr14  chr1  chr12  chr6  chr18  chr2  chr18  chr4  chr7  chr1  chr4  chr4  chr10  chr3  chr17  chr22  chr11  chr4  chr2  chr5  chr6  chr2  chr17  chr18  chr8  chr4  chr17  chr7  chr5  chr18  chr12  chr20  chr12  chr2  chr4  chr2  chr6  chr6  chr1  chr6  chr5  chr4  chr4  chr1  chr9  chr10  chr6  chr6  chr6  chr16  chr12  chr15  chr3  chr17  chr3  chr15  chr1  chr22  chr7  chr12 | 20131441  218861421  52422211  23989613  46864727  76263479  4483453  172520035  218822417  25682525  42003093  48089024  36153481  175875213  48723560  42452442  121529430  75011634  145704447  107809941  103913994  71849992  113429666  59556763  109360426  22566988  152267506  4842353  163951410  71389353  50593241  78445652  20401444  67050493  25871438  10525079  19745638  29827129  176792654  211656506  166189443  50414133  162592328  45213847  43594792  77698009  80322959  67935331  122320038  92645165  9954991  79065113  44644538  64978075  227449615  61374472  9352492  143209546  67547595  37813217  10587056  128675339  114877487  19069745  38419869  12468755  92080999  153868036  34447097  70103822  23251364  46028861  70211175  77027850  32340712  116842794  95223564  191869962  40693110  82016232  16307152 | 20134429  218868404  52427092  23989887  46898455  76265143  4488934  172523947  218828283  25719693  42010995  48965761  36373642  175956526  48853719  42591018  121712303  75162997  146039974  107866217  104154236  72207484  113560554  59655637  109475012  22605948  152343641  4913173  163975178  71402329  50643691  78487498  20436154  67076977  25931155  10539267  19761534  30043909  176814412  211701899  166219476  50442434  162725924  45308068  43616992  77716212  80363910  67961836  122379695  92661242  9966589  79089491  44679339  65005351  227483740  61391604  9380041  143246590  67554974  37850247  10596347  128711239  114936084  19078390  38465078  12498588  92092506  153891240  34464510  70115514  23267921  46047565  70233374  77033270  32351492  116850084  95244115  191871524  40725993  82021827  16313627 | 1.97E-05  4.53E-06  7.39E-06  7.20E-05  1.25E-06  3.61E-05  1.90E-06  9.42E-06  4.95E-06  3.64E-07  4.49E-06  6.71E-08  2.37E-07  4.04E-07  3.24E-07  2.74E-07  3.02E-07  3.41E-07  1.09E-07  5.63E-07  3.59E-07  1.31E-07  3.79E-07  4.57E-07  2.88E-07  9.56E-07  3.07E-07  1.87E-06  9.58E-07  2.14E-06  9.19E-07  5.99E-07  1.08E-06  1.03E-06  8.37E-07  3.15E-06  2.30E-06  3.61E-07  1.53E-06  1.23E-06  2.43E-06  1.05E-06  3.68E-07  9.15E-07  2.26E-06  1.79E-06  1.27E-06  1.30E-06  1.22E-06  1.57E-06  2.37E-06  1.55E-06  1.17E-06  2.72E-06  1.42E-06  3.06E-06  2.45E-06  1.14E-06  3.44E-06  1.02E-06  2.62E-06  8.36E-07  1.88E-06  4.20E-06  1.42E-06  1.83E-06  1.85E-06  5.21E-06  2.44E-06  2.78E-06  5.54E-06  2.11E-06  5.38E-06  7.37E-06  7.00E-06  6.23E-06  8.10E-06  1.03E-05  8.11E-07  1.98E-05  1.69E-05 | nan  nan  nan  nan  0  nan  nan  nan  nan  nan  0  4.73E-07  1.40E-06  2.16E-06  1.67E-06  1.39E-06  1.25E-06  1.19E-06  3.71E-07  1.87E-06  9.09E-07  2.81E-07  7.84E-07  8.47E-07  5.18E-07  1.63E-06  4.73E-07  2.72E-06  1.39E-06  2.81E-06  1.19E-06  7.56E-07  1.22E-06  1.16E-06  8.78E-07  3.05E-06  2.18E-06  3.22E-07  1.34E-06  1.03E-06  2.02E-06  8.23E-07  2.87E-07  6.80E-07  1.66E-06  1.26E-06  8.60E-07  8.62E-07  7.67E-07  9.86E-07  1.42E-06  9.01E-07  6.22E-07  1.36E-06  6.97E-07  1.45E-06  1.15E-06  5.31E-07  1.58E-06  4.65E-07  9.80E-07  3.10E-07  6.28E-07  1.35E-06  4.47E-07  5.54E-07  5.22E-07  1.46E-06  6.81E-07  7.52E-07  1.47E-06  5.51E-07  1.37E-06  1.69E-06  1.45E-06  9.01E-07  1.15E-06  1.40E-06  7.13E-08  1.02E-06  2.53E-07 | nan  nan  nan  nan  nan  nan  nan  nan  nan  nan  nan  0.142005  0.169812  0.186872  0.193792  0.196708  0.241192  0.286614  0.294499  0.300724  0.394901  0.466845  0.48394  0.539173  0.556202  0.58793  0.648727  0.686398  0.688576  0.759634  0.775253  0.791703  0.887491  0.889327  0.952341  1.0317  1.05746  1.12341  1.14077  1.19632  1.20373  1.27829  1.2838  1.34487  1.36243  1.42454  1.47048  1.50765  1.58871  1.58934  1.66581  1.71922  1.88345  2.00338  2.04481  2.11294  2.13616  2.15035  2.18352  2.19865  2.67733  2.7012  3.00125  3.10522  3.17904  3.30278  3.5384  3.57485  3.58124  3.69272  3.78375  3.83514  3.91314  4.35165  4.81395  6.91338  7.05908  7.37505  11.3711  19.4512  66.7342 | 2988  6983  4881  274  33728  1664  5481  3912  5866  37168  7902  876737  220161  81313  130159  138576  182873  151363  335527  56276  240242  357492  130888  98874  114586  38960  76135  70820  23768  12976  50450  41846  34710  26484  59717  14188  15896  216780  21758  45393  30033  28301  133596  94221  22200  18203  40951  26505  59657  16077  11598  24378  34801  27276  34125  17132  27549  37044  7379  37030  9291  35900  58597  8645  45209  29833  11507  23204  17413  11692  16557  18704  22199  5420  10780  7290  20551  1562  32883  5595  6475 |


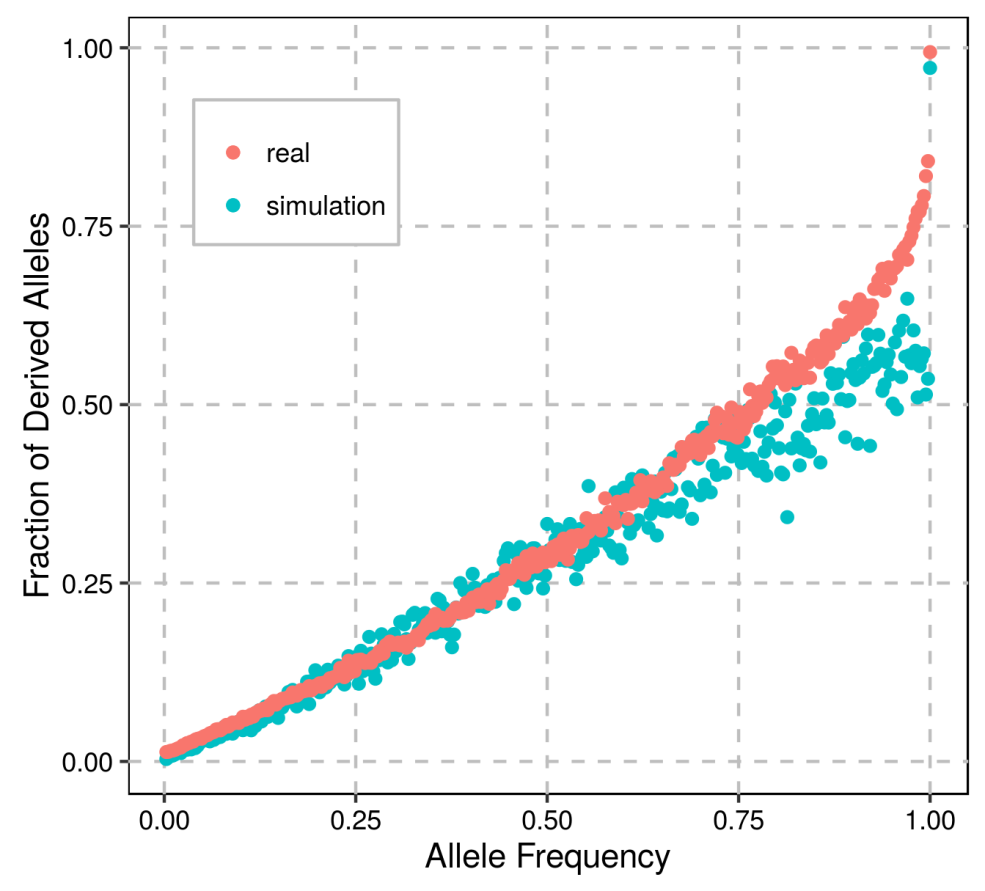


**Supplemental Figure S8**: Fraction of sites that are derived in the Altai Neandertal and segregating in Luhya and Yoruba populations from the 1000G dataset (pink dots) or from simulations under the demographic parameters described in Supplemental Information 1 (blue dots; data from 100 simulated loci of 1Mb). The statistic is stratified by the derive allele frequencies in Africans. Simulations match the pattern seen in the real data well, with the exception of a steep increase at very high frequencies. This discrepancy could be caused by a number of different factors, including sequencing errors that either introduce an ancestral allele in the 1000G data or a derived allele in the Altai Neanderthal, or admixture events. The same pattern and discrepancy is observed with the Denisovan genome (data not shown).

**Supplemental Table S10**: Performance of the Hidden Markov Model for different split times with a selective event starting at half those split times. Effective population sizes (Ne) are assumed constant and time units are reported in 4Ne generations. Selected loci were separated by neutral loci of 100kb. A selected region was defined as a stretch of high posterior probabilities (p ≥ 0.8) for the extended lineage sorting state that was uninterrupted by sites with a low probability (p ≤ 0.2). We note an increase of false positives for split times equal or greater than 4Ne generations and a complete loss of power for split times twice as long.

| **Length cutoff** | **Split Time (in 4Ne)** | **Origin of Selection** | **Selection Coefficient** | **True Positive Rate** | **False Positive Rate** |
| --- | --- | --- | --- | --- | --- |
| >0.025 cM | 0.2 | 0.1 | 0.005 | **0.39** | **0.002** |
| >0.025 cM | 0.6 | 0.3 | 0.005 | **0.62** | **0.056** |
| >0.025 cM | 1 | 0.5 | 0.005 | **0.508** | **0.124** |
| >0.025 cM | 1.4 | 0.7 | 0.005 | **0.228** | **0.102** |
| >0.025 cM | 1.8 | 0.9 | 0.005 | **0.01** | **0.006** |
| >0.025 cM | 2.2 | 1.1 | 0.005 | **0.004** | **0.002** |
| >0.025 cM | 2.6 | 1.3 | 0.005 | **0** | **0.002** |

**Example of commands:**

msms 372 500 -T -N 10000 -t 50 -r 40 100000 -I 3 1 1 370 -ej 0.6 2 3 -ej 6.5 3 1 -SI 0.3 3 0 0 0.0001 -SAA 200 -SAa 100 -Sp 0.5 -Smark –SforceKeep

scrm 372 500 -T -t 50 -r 40 100000 -I 3 1 1 370 -ej 0.6 2 3 -ej 6.5 3 1

**Supplemental Table S11**: Same analysis as Supplemental Table S8 using different probability cutoffs: a selected region was defined as a stretch of high posterior probabilities (p ≥ 0.7) for the extended lineage sorting state that was uninterrupted by sites with a low probability (p ≤ 0.1).

| **Length cutoff** | **Split Time (in 4Ne)** | **Origin of Selection** | **Selection Coefficient** | **True Positive Rate** | **False Positive Rate** |
| --- | --- | --- | --- | --- | --- |
| >0.025 cM | 0.2 | 0.1 | 0.005 | **0.432** | **0.004** |
| >0.025 cM | 0.6 | 0.3 | 0.005 | **0.706** | **0.088** |
| >0.025 cM | 1 | 0.5 | 0.005 | **0.824** | **0.31** |
| >0.025 cM | 1.4 | 0.7 | 0.005 | **0.52** | **0.264** |
| >0.025 cM | 1.8 | 0.9 | 0.005 | **0.048** | **0.012** |
| >0.025 cM | 2.2 | 1.1 | 0.005 | **0.014** | **0.016** |
| >0.025 cM | 2.6 | 1.3 | 0.005 | **0.006** | **0.004** |

# REFERENCES

The 1000 Genomes Project Consortium. 2012. An Integrated Map of Genetic Variation from 1,092 Human Genomes. *Nature* **491**: 56–65.

Aken BL, Ayling S, Barrell D, Clarke L, Curwen V, Fairley S, Fernandez Banet J, Billis K, García Girón C, Hourlier T, et al. 2016. The Ensembl gene annotation system. *Database* baw093. doi: 10.1093/database/baw093.

Anders S, Huber W. 2010. DESeq: Differential Expression Analysis for Sequence Count Data. *Genome biology* **11**: R106. doi**:** 10.1186/gb-2010-11-10-r106.

Danecek P, Auton A, Abecasis G, Albers CA, Banks E, DePristo MA, Handsaker RE, Lunter G, Marth GT, Sherry ST, et al. 2011. The Variant Call Format and VCFtools. *Bioinformatics* **27**: 2156–2158.

Derrien T, Johnson R, Bussotti G, Tanzer A, Djebali S, Tilgner H, Guernec G, Martin D, Merkel A, Knowles DG, et al. 2012. The GENCODE v7 Catalog of Human Long Noncoding RNAs: Analysis of Their Gene Structure, Evolution, and Expression. *Genome Research* **22**: 1775–1789.

Dutheil JY, Munch K, Nam K, Mailund T, Schierup MH. 2015. Strong Selective Sweeps on the X Chromosome in the Human-Chimpanzee Ancestor Explain Its Low Divergence. *PLoS Genetics*, **11**(8). http://doi.org/10.1371/journal.pgen.1005451

Ewing G, Hermisson J. 2010. MSMS: A coalescent simulation program including recombination, demographic structure and selection at a single locus. *Bioinformatics* **26**: 2064–2065. http://doi.org/10.1093/bioinformatics/btq322

Harris RS. 2007. Improved pairwise alignment of genomic DNA. Ph.D. Thesis, The Pennsylvania State University.

Hinch AG, Tandon A, Patterson N, Song Y, Rohland N, Palmer CD, Chen GK, Wang K, Buxbaum SG, Akylbekova EL, et al. 2011. The Landscape of Recombination in African Americans. *Nature* **476**: 170–175.

Kong A, Thorleifsson G, Gudbjartsson DF, Masson G, Sigurdsson A, Jonasdottir A, Walters GB, Jonasdottir A, Gylfason A, Kristinsson KT, et al. 2010. Fine-Scale Recombination Rate Differences between Sexes, Populations and Individuals. *Nature* **467**: 1099–1103.

Locke DP Hillier LW Warren WC Worley KC Nazareth LV Muzny DM Yang SP Wang ZY Chinwalla AT Minx P et al. 2011. Comparative and demographic analysis of orang-utan genomes. *Nature* **469**: 529-533.

Messer PW. 2013. SLiM: Simulating evolution with selection and linkage. *Genetics*. http://doi.org/10.1534/genetics.113.152181.

Mikkelsen TS, Hillier LW, Eichler EE, Zody MC, Jaffe DB, Yang SP, Enard W, Hellmann I, Lindblad-Toh K, Altheide TK et al. 2005. Initial sequence of the chimpanzee genome and comparison with the human genome. *Nature* **437**: 69-87.

Munch K, Mailund T, Dutheil JY, Schierup MH. 2014. A fine-scale recombination map of the human-chimpanzee ancestor reveals faster change in humans than in chimpanzees and a strong impact of GC-biased gene conversion. *Genome Research* **24**: 467–474. http://doi.org/10.1101/gr.158469.113

Nam K, Munch K, Hobolth A, Dutheil JY, Veeramah KR, Woerner AE, Hammer MF, Great Ape Genome Diversity Project, Mailund T, et al. 2015. Extreme Selective Sweeps Independently Targeted the X Chromosomes of the Great Apes. *Proceedings of the National Academy of Sciences* **112**: 6413–6418.

Prüfer K, Muetzel B, Do HH, Weiss G, Khaitovich P, Rahm E, Pääbo S, Lachmann M, Enard W. 2007. FUNC: A Package for Detecting Significant Associations between Gene Sets and Ontological Annotations. *BMC bioinformatics* **8**: 41.

Prüfer K, Munch K, Hellmann I, Akagi K, Miller JR, Walenz B, Koren S, Sutton G, Kodira C, Winer R et al. 2012. The bonobo genome compared with the chimpanzee and human genomes. *Nature* **486**: 527-531.

Prüfer K, Racimo F, Patterson N, Jay F, Sankararaman S, Sawyer S, Heinze A, Renaud G, Sudmant PH, de Filippo C, et al. 2014. The Complete Genome Sequence of a Neanderthal from the Altai Mountains. *Nature* **505**: 43–49.

Racimo F. 2016. Testing for ancient selection using cross-population allele frequency differentiation. *Genetics* **202**: 733–750. http://doi.org/10.1534/genetics.115.178095

Racimo F, Kuhlwilm M, Slatkin M. 2014. A test for ancient selective sweeps and an application to candidate sites in modern humans. *Molecular Biology and Evolution* **31**: 3344–3358. http://doi.org/10.1093/molbev/msu255

Sankararaman S, Mallick S, Dannemann M, Prüfer K, Kelso J, Pääbo S, Patterson N, Reich D. 2014. The genomic landscape of Neanderthal ancestry in present-day humans. *Nature* **507**: 354–357.

Scally A, Dutheil JY, Hillier LW, Jordan GE, Goodhead I, Herrero J, Hobolth A, Lappalainen T, Mailund T, Marques-Bonet T et al. 2012. Insights into hominid evolution from the gorilla genome sequence. *Nature* **483**: 169-175.

Speir ML, Zweig AS, Rosenbloom KR, Raney BJ, Paten B, Nejad P, Lee BT, Learned K, Karolchik D, Hinrichs AS, et al. 2016. The UCSC Genome Browser Database: 2016 Update. *Nucleic Acids Research* **44**: D717–725.

Staab PR, Zhu S, Metzler D, Lunter G. 2014. Scrm: Efficiently simulating long sequences using the approximated coalescent with recombination. *Bioinformatics* **31**: 1680–1682. http://doi.org/10.1093/bioinformatics/btu861
